# Cytokeratin 18 Is Not Required for Morphogenesis of Developing Prostates but Contributes to Adult Prostate Regeneration

**DOI:** 10.1155/2013/576472

**Published:** 2013-12-30

**Authors:** Chenlu Zhang, Yanjing Guo, Jian Cui, Helen He Zhu, Wei-Qiang Gao

**Affiliations:** ^1^State Key Laboratory of Oncogenes and Related Genes, Stem Cell Research Center, Renji Hospital, 160 Pu Jian Road, Shanghai Jiao Tong University School of Medicine, Shanghai 200127, China; ^2^Med-X Research Institute, Shanghai Jiao Tong University, Shanghai 200030, China

## Abstract

Cytokeratin 18 (CK18) is a key component of keratin-containing intermediate filaments and has long been used as a classic luminal cell marker in prostatic tissue. However, the *in vivo* function of CK18 in prostate is not known so far. We reported in this study, unexpectedly, that deletion of CK18 in a mouse model did not affect the morphological or the histological structures of adult prostate, as the CK18 knockout prostate displayed a normal glandular ductal structure, branching pattern, and composition of both luminal and basal cells. However, CK18 loss compromised the regenerative tubular branching in dorsolateral prostate after castration and androgen replacement. Therefore, in contrast to its importance as luminal cell marker, CK18 is dispensable for the prostate morphogenesis but contributes to adult prostate regeneration.

## 1. Introduction

The glandular epithelium of prostate is composed of three types of differentiated cells: luminal cells, basal cells, and rare neuroendocrine cells. Different keratins are preferentially expressed in specific prostatic epithelial cells and are routinely used as markers for respective epithelial lineages. The rectangular lumen-lining luminal cells constitute the major part of the prostate epithelial and express cytokeratin 18 (CK18), cytokeratin 8 (CK8), and androgen receptor (AR) [1], while the small and oblong basal cells frequently found adjacent to the basement membrane are positive for p63, CK5, and CK14 but do not express CK8, CK18, or AR. Deletion of basal cell maker p63 in mouse model led to absence of basal cells in prostate explants [2–4].

CK18 is a type II cytokeratin and usually coexists with type I cytokeratin CK8 to produce keratin-containing intermediate filaments [5]. Keratin intermediate filaments form cage-like structures around the nucleus, which play an essential role in the maintenance of nuclear integrity. Therefore, CK18 acts as an important scaffold protein in response to external stresses. It is also involved in regulation of cellular processes including apoptosis, mitosis, and cell cycle [5]. Although homozygous CK18 knockout mice are viable and fertile, liver disorders with CK8-positive aggregates are found in aged CK18^−/−^ animals [6]. In contrast to the importance of CK18 as a luminal cell marker, its *in vivo* function in the prostate morphogenesis and regeneration remains unexplored so far.

The mouse prostate development initiates from the urogenital sinus (UGS) [7]. The prostatic epithelium buds out around embryonic day 17.5 (E17.5) and elongates and branches after birth. The branching morphogenesis almost completes at postnatal week two but fully matures by 60 to 90 days [7, 8]. The adult prostate is composed of three symmetrical lobes: the anterior prostate (AP), ventral prostate (VP), and dorsolateral prostate (DLP) [9]. Prostate development, branching, and maintaining rely on serial androgen release and response. Deprivation of androgen induces intensive atrophy of prostate lobes, while androgen replacement efficiently stimulates prostate regeneration [10, 11]. This atrophy-regeneration process can be repeated around thirty times in rats in support of the notion that stem-like cells exist in castration-resistance prostate cells [10].

We performed in this study morphological and histological investigation of CK18^−/−^ prostate in both physiological and regenerative conditions. We reported here, against our original hypothesis, that CK18 may be required for the morphogenesis and regeneration of prostate; deletion of CK18 in a mouse model did not affect the normal morphology and histology of prostate. However, loss of CK18 indeed compromised the branching of regenerative DLP.

## 2. Materials and Methods

### 2.1. Experimental Animals

CK18^−/−^ mice were introduced from Jackson Laboratory originated deposited by Dr. M. Bishr Omary. Genotyping of CK18^−/−^ mice is performed as previously reported in [12]. All mice were housed in experimental animal center at Renji Hospital, Shanghai Jiaotong University, in pathogen-free environment with controlled temperature and humidity. Experimental procedures follow the guidelines and recommendations from Institutional Laboratory Animal Use and Care Committee (IACUC). For genotyping, 2 mm tail sample was obtained for genomic DNA extraction. 150 uL 25 mM NaOH/0.2 mM EDTA was added to the tail sample and kept at 98°C for 1 hour, followed by neutralization with 150 uL of 40 mM Tris-HCl (pH = 5.5). After a brief centrifugation, 2 *μ*L of supernatant was used for PCR reaction. The genotyping primers we used are 5′-AAG GAA TCC AGG AAG GGA GA-3′; 5′-AGC CCC GGA CTT ACT TGA CT-3′, and 5′-GCC AGA GGC CAC TTG TGT AG-3′. Thermal cycling parameters included (i) an denaturation at 95°C for 5 min; (ii) 30 cycles of denaturation at 95°C for 30 s, annealing at 60°C for 1 min, and extension at 72°C for 1 min; and (iii) a final extension at 72°C for 5 min. The PCR products for mutant and wild-type allele are 187 bp and 312 bp, respectively.

### 2.2. Hematoxylin and Eosin (H&E) Staining

Prostate samples were fixed in 4% paraformaldehyde, dehydrated and paraffin embedded. After that, the paraffined prostates were cut at 5 *μ*m thickness on a microtome. Slides were stained with hematoxylin and eosin following standard protocols. Multiple fields were selected for each H&E-stained section to compare the differences in prostate.

### 2.3. Immunoblotting

100 **μ**g protein samples extracted from both wild-type and CK18^−/−^ knockout prostates were used for electrophoresis in 10% sodium dodecyl sulfate polyacrylamide gel. Antibodies against CK18 (Abcam) were used as primary antibody.

### 2.4. Castration and Androgen Replacement

Four 2-month-old male mice of each genotype are used in our experiment. After anesthesia with avertin, the testes and epididymides were completely removed. The distal end of the spermatic cord was ligated with suture. 14 days after castration, a 0.8 cm sustained-release capsule of stanolone (30–40 mg, Sigma) was implanted subcutaneously. The mice were sacrificed for prostate collection 14 days later.

### 2.5. Prostate Microdissection

The separated prostate was moderately digested in 10 mg/mL collagenase for 10 min. After digestion, microdissection was performed to visualize the individual ductal networks of prostate with fine forceps. All specimens were observed and photographed with microdissection microscope (Nikon SMZ800) for branch and tip quantification.

### 2.6. Immunofluorescence Staining

Immunofluorescence staining was performed with 8 um cryosections. After fixation in 4% PFA at room temperature for 5 min, sections were immerged in sodium citrate solution (10 mM, pH = 6.0) and microwaved for antigen retrieval for 5 min. Sections were subsequently treated with 0.5% triton for 10 min, followed by PBS wash and blocking with 10% normal goat serum (NGS) for 1 hour at room temperature. Specimens were then incubated with the primary antibodies overnight at 4°C. Primary antibodies were washed at least three times with PBS containing 1% NGS. After washing, secondary antibodies conjugated with Alexa-488 or 594 (Jackson Laboratory) were added to the specimens and incubated for 1 hour at room temperature. Secondary antibody was washed with PBS containing 1% NGS 3 times. Slides were then mounted with DAPI containing mounting medium from Vector Laboratories. We used the followed primary antibodies in the immunofluorescence staining experiments: anti-p63 (Santa Cruz, sc-8431), anti-CK5 (Epitomics, 1988-1), anti-CK8 (Covance, MMS-162P), anti-CK8 (Epitomics, 2032-1), and anti-CK18 (Epitomics, 3258-1).

## 3. Results

### 3.1. H&E Staining Shows No Changes in Histology of CK18^−/−^ Mouse Prostates

**Figure 1:**
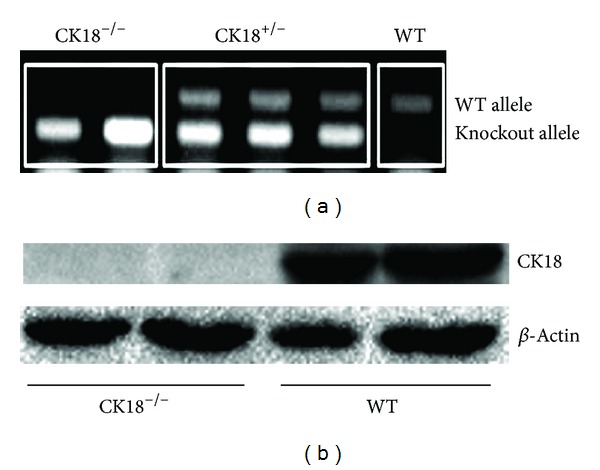
CK18 deletion in prostate from CK18^−/−^ mouse is verified by PCR and immunoblotting. (a) CK18 null allele and wild-type (WT) allele were detected by PCR analysis of tail samples. (b) Immunoblotting verifies ablation of CK18 expression in the CK18^−/−^ mouse prostate. Two mice of each genotype are used.

**Figure 2:**
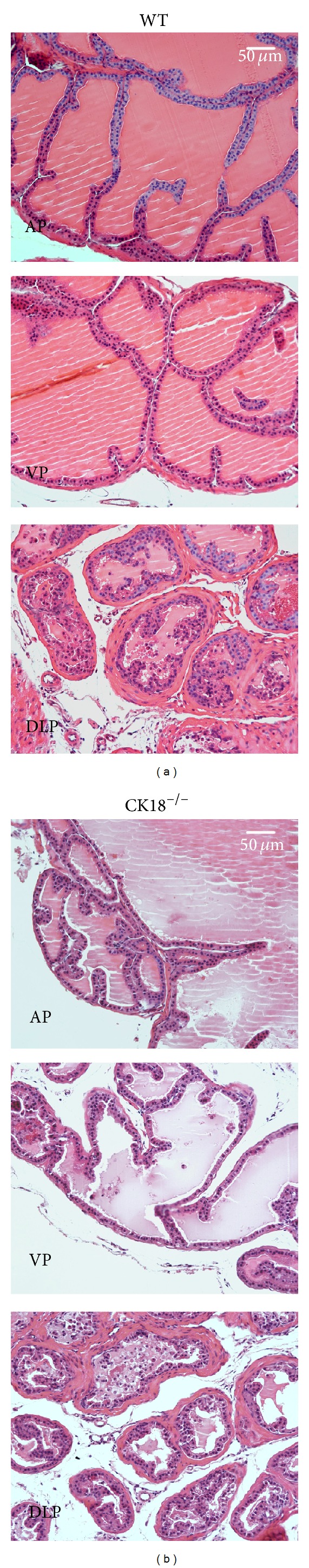
H&E staining displays no histological difference between WT and CK18^−/−^ mouse prostates. Representative H&E staining of AP, VP, and DLP prostates from WT (a) and CK18^−/−^ (b) mice, shows normal histology of CK18^−/−^ prostates. Images were taken by Nikon light microscope (Nikon Eclipse Ti) with 200x magnification. Scale bars are 50 *μ*m for each related row.

To investigate the *in vivo* function of CK18 in prostate morphogenesis, we made use of CK18^−/−^ mice. Genomic PCR detected the null alleles in CK18^−/−^ mice (Figure 1(a)). Immunoblotting further confirmed a complete CK18 deletion in prostates collected from knockout animals (Figure 2(a)). In order to assess the impact of CK18 deletion on the histology of developing prostate, we performed H&E staining of prostates from mutant and control mice at different postnatal development stages. Surprisingly we did not find any difference in gross appearance and histological structure between CK18^−/−^ and WT prostates. As shown in Figure 2, size of prostate from 8-week-old CK18^−/−^ mice was comparable to WT. H&E staining of mutant prostate AP, VP, and DLP did display a normal glandular structure and branching pattern (Figure 2). We examined carefully the prostate of 1-week-old and 4-week-old mutant mice as well, but no histological changes were detected (data not shown).

### 3.2. Both Luminal and Basal Cell Compartments Are Detected in the CK18^−/−^ Mouse Prostate

**Figure 3:**
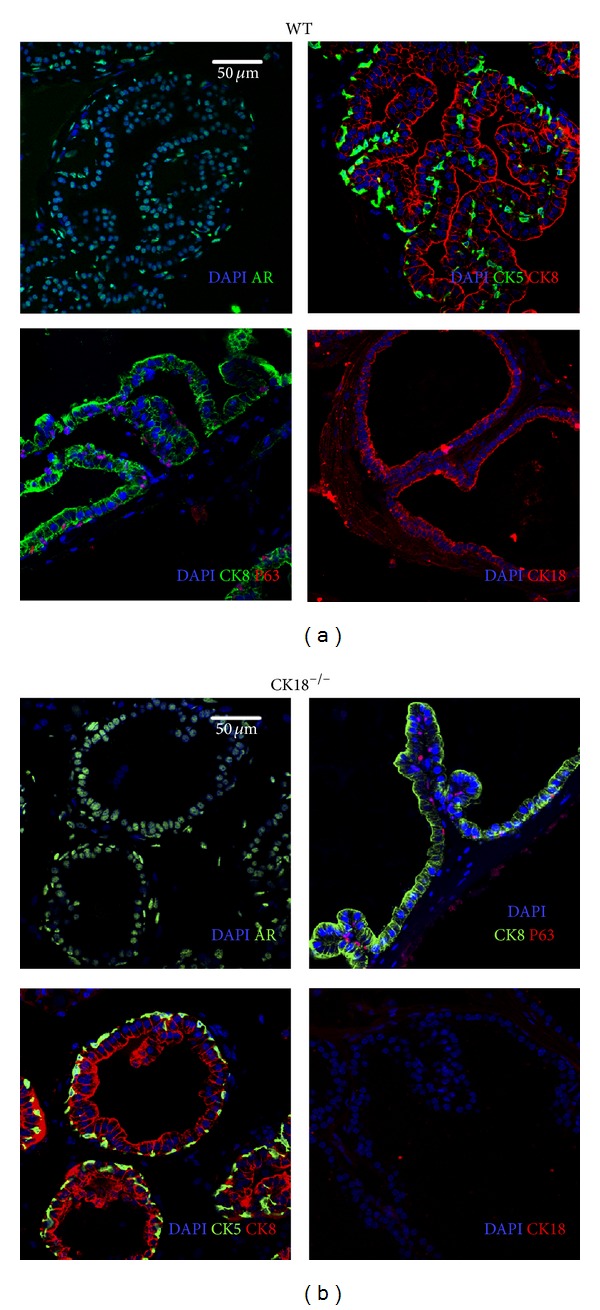
Normal basal and luminal cell compartments in CK18^−/−^ prostates. Immunofluorescence staining of prostate frozen sections displays similar luminal and basal cell compartments in WT (a) and CK18^−/−^ (b) prostates. Luminal cells are positive for AR, CK8, and CK18, while the basal cells are CK5 and P63 positive. At least 4 mice were sacrificed in each group for prostate collection, sectioning, and staining. Multiple fields were thoroughly examined under a microscope. Representative images are shown. Pictures were taken using a Nikon fluorescent microscope (Nikon Eclipse Ti) with 400x magnification. Scale bars are 50 *μ*m for each related row.

CK18 is a frequently used luminal cell marker. To investigate if CK18 loss affects the luminal cell compartment, we performed immunofluorescence staining on mutant and WT prostates using well-characterized markers for luminal cells and basal cells. As expected, no expression of CK18 was detected from prostates of mutant mice (Figure 3). P63 and CK5 are selectively expressed in basal cells, while CK8, CK18, and AR are preferentially expressed in luminal cells. As shown in Figure 3, both luminal and basal cells were detected in CK18^−/−^ prostates, with comparable numbers to WT controls, implicating that CK18 is not required for luminal cell differentiation in the prostate.

### 3.3. CK18 Is Dispensable for Prostate Branching

**Figure 4:**
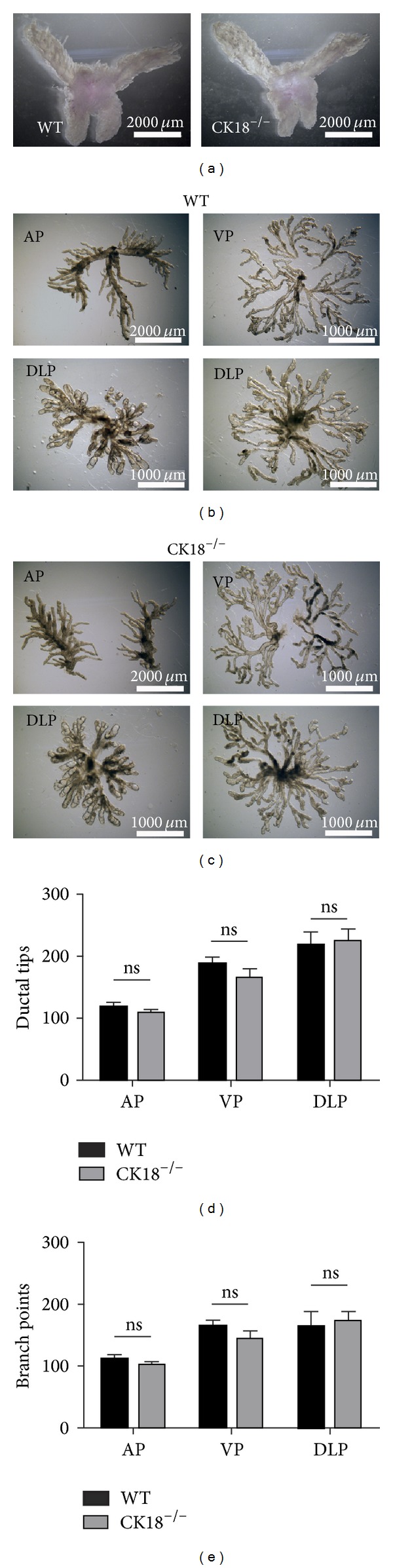
CK18 is dispensable for prostate branching. (a) The gross anatomic appearance of WT and CK18^−/−^ prostates with AP, VP, and DLP. Scale bars are 2000 *μ*m. (b, c) The microdissection images display the ductal networks of prostate in left or right lobe of AP, VP, and DLP from WT and CK18^−/−^ mice (a). Four mice of each group are used in the experiment. Only representative pictures are shown. Scale bar for AP image is 2000 *μ*m, in VP and DLP images is 1000 *μ*m. (d, e) Bar graph shows no significant differences of ductal tips and branch points in AP, VP, and DLP between WT and of CK18^−/−^ prostates (*n* = 4).

Tubular morphogenesis of the mouse prostate completes at two months after birth. The maturation of AP, DP, and DLP accomplishes by then (Figures 4(a) and 4(b)). We further examined the function of CK18 in prostate tubular branching. The complex and extended prostatic ductal networks of all three lobes can be readily seen under microscope after brief digestion and careful microdissection (Figures 4(b) and 4(c)). The ductal tips and branching points were counted in each group to quantify the tubular formation. Statistical analysis showed no significant difference of ductal tips and branching points between mutant and WT groups, indicating normal tubular morphogenesis in CK18^−/−^ prostates.

### 3.4. CK18 Deletion Reduces the Branching of DLP during Prostate Regeneration Induced by Androgen Deprivation and Replacement

**Figure 5:**
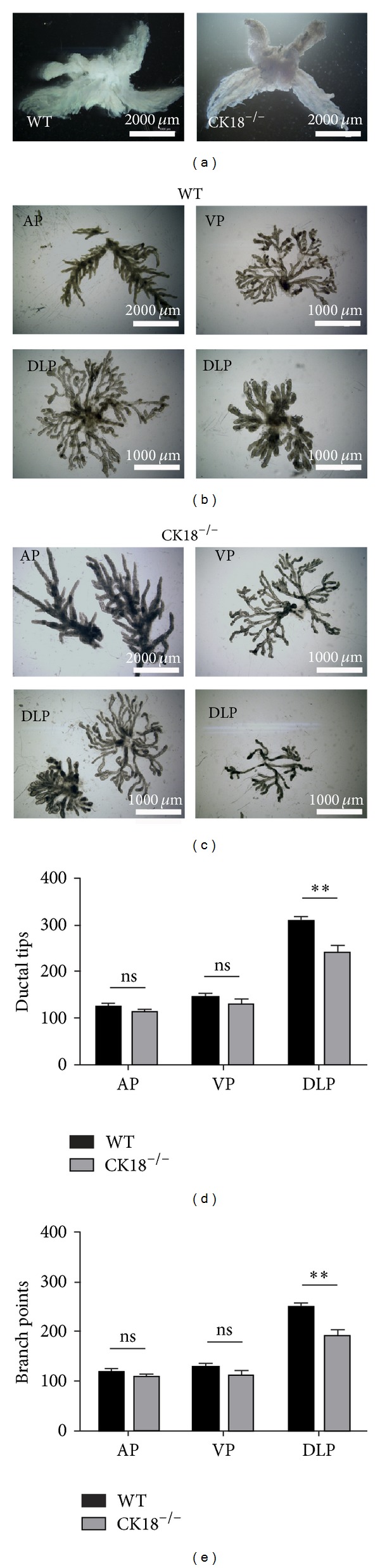
CK18 is required for DLP branching in prostate regeneration. (a) Prostates are carefully dissected after castration and androgen replacement. The gross anatomic appearance reveals no obvious difference between WT and CK18^−/−^ prostates. Scale bars are 2000 *μ*m. (b, c) The images of microdissected prostates from WT (a) and CK18^−/−^ (b) mice after castration and androgen replacement show compromised branching pattern in DLP but not in VP and AP of CK18^−/−^ prostate. Four mice of each group are used in the experiment. Only representative pictures are shown. The scale bar in AP image is 2000 *μ*m; scale bars in VP and DLP images are 1000 *μ*m. (d, e) Bar graphs show significantly reduced umbers of ductal tips (mean ± SEM) and branch points in CK18^−/−^ DLP (mean ± SEM, **denotes *P* < 0.02).

Androgen deprivation induces atrophy of prostate lobes, while administration of androgen efficiently stimulates prostate regeneration in rodents. To investigate if CK18 plays a role in a stress scenario such as prostate regeneration, we subjected both mutant and WT animals to castration and androgen replacement. Two-month-old mice from each group were used for castration. 14 days later, the castrated mice were given androgen by subcutaneous implants for another 14 days and then were sacrificed for prostate collection. Regenerated CK18^−/−^ prostates displayed similar gross anatomic appearances to control (Figure 5(a)). However, when we carefully microdissected each regenerated prostatic lobes, we did notice compromised tubular restoration in DLP from CK18^−/−^ mice (Figures 5(b) and 5(c)). Consistently, quantitative data showed significant reduction of both ductal tips and branch points in CK18^−/−^ DLP compared to WT control, suggesting that CK18 does play a role in the prostate in response to stress and contributes to prostate regeneration.

## 4. Discussion

The morphogenesis of developing prostate is governed by extracellular signal molecules and intracellular genetic programs [13–16]. For example, sonic hedgehog (Shh) pathway inhibits epithelial ductal branching of the prostate as reduced number of epithelial ducts was found in Shh-treated postnatal day 2 rat ventral prostate [17–19]. In contrast, castrated prostates could not regain normal size and structure after androgen replacement when the hedgehog pathway is blocked [20]. BMPs including BMP4 and BMP7 are additionally shown to be negative regulators in prostate ductal budding and branching [21, 22]. Basal-cell-specific transcriptional factor p63 is indispensable for prostate development. UGS of p63^−/−^ mice is only able to generate prostatic structures with luminal and neuroendocrine cells but fails to give rise to any basal cells upon transplantation in renal capsule [4, 23]. CK18 is preferentially expressed in prostate luminal cells and has long served as luminal cell marker. We examined the morphology and histology of CK18^−/−^ prostates in this study to investigate the *in vivo* function of CK18 in prostate. To our surprise, CK18 deletion did not cause any morphological or histological change in homozygous *ck18* knockout mice. However, when the prostate is under regenerative stress after castration and androgen replacement, CK18 ablation leads to partially compromised branching of DLPs.

CK18 and CK8 are the most abundant coexpressed keratins in mammals [24]. In the liver, CK18 and CK8 comprise the hepatocellular keratin, while CK18, CK8, and CK19 are the major keratins in hepatobiliary ductal cells [25]. Mutations of CK18 are associated with predisposition to subacute injury, apoptosis, and fibrosis of liver, possibly due to tempered CK18 phosphorylation and instability of filament structure [25–27]. In epidermis, keratins are the major proteins constituting the cytoskeleton and protecting cell from mechanistic stress. CK5 and CK14 are often coexpressed as specific pairs in basal cell compartment. Deletion of CK14 in mice dramatically increases CK15 is that rarely expressed in basal cell [28]. Moreover, CK15 is able to reconstitute CK14 to form the keratin networks with CK5, implicating a redundant function of CK14 and CK15 *in vivo*. CK1 and CK10 are keratin pairs in suprabasal layer of epidermis. Similar phenomenon was also observed in CK10^−/−^ mice, in which upregulation of basal keratins 5 and 14 permits the normal epidermal differentiation of CK10^−/−^ mice [29]. Redundancy among CK6*α* and CK6*β*4, CK4 and CK6, and CK19 and CK18 was further identified in the epidermis [30–32]. The result we show here that CK18 deletion barely affects prostate morphogenesis is probably also caused by upregulation of redundant cytokeratin. Increased CK19 expression is detected in CK18^−/−^ livers and double knockout of CK18 and CK19 led to much severe phenotypes including trophoblast fragility and severe early development defects [6, 32–34]. Therefore, we propose that CK19 which normally is not expressed in luminal cells may be upregulated in CK18^−/−^ prostates. However, embryonic lethality in the double knockout animals prevents us from the *in vivo* study of CK18 and CK19 redundancy. It is noteworthy that we did observe defects in CK18^−/−^ DLP branching morphogenesis when the prostate undergoes great stress during regeneration. Further analysis of expression level of other keratins in CK18^−/−^ prostate and experiment approaches like additional knocking down of relevant keratins will facilitate the identification of CK18 redundant protein in prostatic morphogenesis and regeneration.

## 5. Conclusion

In summary, our data suggest that although CK18 is an important marker for luminal cells, it has limited impact on the histology and ductal formation of the prostatic epithelium possibly due to functional redundancy of other keratins. However, in stress conditions such as regeneration process, deletion of CK18 partly compromises the branching and full recovery of DLP.
